# Ultrafast bold fMRI using single-shot spin-echo echo planar imaging

**DOI:** 10.4103/0971-6203.48719

**Published:** 2009

**Authors:** Saïd Boujraf, Paul Summers, Faouzi Belahsen, Klaas Prüssmann, Spyros Kollias

**Affiliations:** 1 Department of Biophysics and Clinical MRI Methods, Faculty of Medicine and Pharmacy, University of Fez, Fez, Morocco; 2 Institute of Neuroradiology, University Hospital of Zurich, Zurich, Switzerland; 3 Neurology Department, University Hospital of Fez, Fez, Morocco; 4 Institute of Biomedical Engineering, ETH, Zurich, Switzerland

**Keywords:** Bold-fMRI, parallel imaging, sense, spin-echo echo planar imaging, Tesla

## Abstract

The choice of imaging parameters for functional MRI can have an impact on the accuracy of functional localization by affecting the image quality and the degree of blood oxygenation-dependent (BOLD) contrast achieved. By improving sampling efficiency, parallel acquisition techniques such as sensitivity encoding (SENSE) have been used to shorten readout trains in single-shot (SS) echo planar imaging (EPI). This has been applied to susceptibility artifact reduction and improving spatial resolution. SENSE together with single-shot spin-echo (SS-SE) imaging may also reduce off-resonance artifacts. The goal of this work was to investigate the BOLD response of a SENSE-adapted SE-EPI on a three Tesla scanner. Whole-brain fMRI studies of seven healthy right hand-dominant volunteers were carried out in a three Tesla scanner. fMRI was performed using an SS-SE EPI sequence with SENSE. The data was processed using statistical parametric mapping. Both, group and individual subject data analyses were performed. Individual average percentage and maximal percentage signal changes attributed to the BOLD effect in M1 were calculated for all the subjects as a function of echo time. Corresponding activation maps and the sizes of the activated clusters were also calculated. Our results show that susceptibility artifacts were reduced with the use of SENSE; and the acquired BOLD images were free of the typical quadrature artifacts of SS-EPI. Such measures are crucial at high field strengths. SS SE-EPI with SENSE offers further benefits in this regard and is more specific for oxygenation changes in the microvasculature bed. Functional brain activity can be investigated with the help of single-shot spin echo EPI using SENSE at high magnetic fields.

## Introduction

Functional magnetic resonance imaging (fMRI) based on a blood oxygen-level dependent (BOLD) contrast mechanism, has become a powerful tool for neuroscientists to investigate the functional organization of the human brain.[1–4] Gradient echo planar imaging (GE-EPI) at a resolution of 64 × 64 is the most commonly used technique for this purpose. This approach has proven to be robust in the investigation of human brain function at 1.5 Tesla, as it is sensitive to the BOLD effect without being prone to subject motion artifacts and tissue pulsations.[5–7]

However, these techniques require excellent gradient performance, leaving the images subject to blurring and warping, caused by imperfect gradient performance, T*_2_, and off-resonance effects. Increasing the static field strength B_0_ only enhances the sensitivity of GE-EPI scans to blurring and wrapping caused by short T*_2_ and off-resonance effects. Although this may be partially accommodated for by reducing the echo times, doing so, will place an additional demand on the gradient performance.[5–7]

SE-EPI is less prone to these artifacts.[8,9] Recently, SE-EPI experiments were suggested on both 1.5 Tesla and 3 Tesla systems to obtain spin-echo (SE) fMRI data from both the brain and spinal cord. In contrast to what is usually expected of the BOLD effect, the SE fMRI data do not show any signal changes that approach zero as the echo time (TE) approaches zero.[8,9]

A second approach has been introduced to reduce both the blurring and warping seen in SS-EPI, which is the combination of EPI with recently introduced parallel imaging techniques.[10–17] These techniques use the spatial differences in sensitivity profiles of the individual channels in detector arrays[11,17] to reconstruct MR images from reduced field-of-view (FOV) data. For a given resolution, this under-sampling strategy could be used to reduce image artifacts by shortening the data acquisition window,[11,17] or to reduce gradient switching rates.[13]

Parallel imaging techniques have shown great promise in applications such as cardiac imaging,[14,15] angiography[16] and diffusion-weighted imaging.[12] However, the benefit for single-shot fMRI has not yet been completely demonstrated.

The preliminary demonstrations of SENSE fMRI showed that a twofold increase in scan speed can be achieved at a relatively small reduction in signal stability.[17] Moreover, the selection of the MRI method may however, have an impact on localization accuracy by affecting image quality and the degree of BOLD contrast achieved.[18] In order to reduce artifacts and improve spatial resolution in fMRI,[17] we have examined a combination of SENSE with single-shot spin-echo EPI imaging. This combination should offer reduced off-resonance artifacts and should improve the anatomical localization and detectability of the functional signal.

The goal of this work was to investigate the BOLD response of a SENSE-adapted single-shot spin-echo EPI using a motor task on a 3 Tesla scanner and optimize the sequence parameters for detection of activation-induced signals.

We scrutinized the ability of this approach to localize the motor-sensory activation using a simple hand-clenching task.

## Materials and Methods

### Background

Sensitivity encoding (SENSE) is a parallel imaging method that uses the spatial inhomogeneity in the sensitivity of receiver coil arrays to reduce the number of phase-encoding steps used in conventional imaging approach. This reduction is critical in decreasing the scan time and improving the spatial and temporal resolutions. To perform the unfolding of aliased single-coil images in image space, the SENSE approach requires a set of calibration images to define coil sensitivities throughout the desired FOV.[11] The number of coil elements involved in the measurement constitutes the upper limit for the reduction of the number of phase-encoding steps acquired.[11]

The signal-to-noise ratio (SNR) in the SENSE imaging approach is defined by the following relationship:[11–18]

(1) $$\begin{array}{c} {\mathrm{SNR}}^{\mathrm{SENSE}}=\frac{{\mathrm{SNR}}^{\mathrm{Full}}}{g\sqrt{R}} \end{array}$$

where SNR^SENSE^ is the SNR when using the SENSE imaging approach, SNR^Full^ is the SNR when using the conventional imaging approach, g is the local geometry factor, and R denotes the factor by which the number of samples is reduced with respect to the conventional imaging approach.

Notably, both R and g are always greater or equal to the unit[11–13] with the effect that the SNR^SENSE^ in SENSE imaging is less than that of the equivalent image acquired without SENSE; the mathematical expressions of g and R are given in the reference.[11]

The combination of SENSE with single-shot EPI methods can be exploited to increase the matrix size while the total length of the readout period is kept constant, or to shorten the acquisition window by reducing the echo train length.

To achieve good sensitivity of the BOLD signal in fMRI measurement, a relatively long echo time is used (TE ≥ 55 ms at 1.5 Tesla and TE ≥ 40 at 3 Tesla)[8] at higher static field strengths, however, a shorter T*_2_ relaxation times are limiting factor. SENSE can help in shortening the scan time when decreasing the echo times while moving to higher field strengths.

Alternatively, a general increase in spatial resolution can be achieved in areas with extremely short T*_2_,[19] and typical EPI artifacts such as image distortions can be clearly reduced by the use of faster k-space traversal.[20] Recent studies have shown the possibility of combining the SENSE imaging approach with EPI techniques when operating at a static field of 3 Tesla to avoid the typical distortion occurring in typical EPI images.[21–23] Achieving images free of distortions and artifacts with high resolution is not enough in fMRI studies in ultra fast modes, because the most important issue is to achieve images with enough sensitivity to the BOLD signal induced in the brain by its involvement in a given function. Thus, the reproducibility of the localization of the activation map corresponding to a given brain activity is critical within that respect for whatever imaging technique used. Recent studies using SENSE in fMRI studies have demonstrated this possibility at low spatial resolution with lower SENSE reduction factors.[13–15]

We have used single shot spin echo EPI combined with SENSE at high resolution in our study.

### MRI hardware

All the experiments were carried out in a 3 Tesla scanner (Gyroscan Intera, Philips Medical Systems, Best, Holland) using the body coil for rf-excitation and an eight-element head coil (MRI Devices Corporation, Waukesha WI, USA) connected to six independent channels. The maximum gradient strength is 30mT/m with a maximum slew rate of 150 mT/ms.

### fMRI protocol

Seven healthy right-handed volunteers were scanned (four males and three females, average age 33 ± 6 years) using a paradigm designed to produce activation in the motor and sensory regions of the brain.

The subjects performed a self-paced (∼1.5Hz), simple motor task with the dominant hand for 30 seconds, alternating with rest in a block design for three minutes, resulting in the collection of 72 time points. The functional experiment was performed twice for each echo time. The volunteers were instructed and trained before the scan session, and were reminded of the instructions immediately prior to each fMRI scan.

### Imaging protocol

Following a three-plane localizer scan, a full-FOV reference scan was performed with the body coil and each array element.[11]

Due to its large FOV, which comprised the FOV of all subsequent SENSE imaging protocols and appropriate interpolations, a 3D spoiled gradient echo sequence was used as the reference scan. The measurement parameters were as follows: matrix size 64 × 64 × 80 slices, FOV 270 mm, slice thickness 3.75 mm, TE 1.53 ms, TR 8 ms, flip angle 7°, and an acquisition time of 2 min, 36s. A reference scan was acquired once for each subject. The generation of sensitivity maps and SENSE reconstruction was performed at the scanner console using standard routines (Philips Medical Systems).

The functional experiments were conducted using single-shot SE-EPI imaging protocols with a SENSE reduction factor of *R* = 2.75, and a high spatial resolution (128 × 128) acquisition. The other scan parameters were defined as follows: TR = 2400 ms, FOV = 225 mm, flip angle of 90°, and 22 slices, to facilitate whole brain coverage; the slice thickness was 4 mm with an interslice gap of 0.4 mm. Scan duration was 3 min when 72 volumes were acquired. Likewise, the repetition time (TR) was kept constant at 2400 ms while the echo time was varied from 30 to 60 ms at increments of 5ms. Using the same paradigm has thus, yielded seven fMRI measurements with echo times of 30, 35, 40, 45, 50, 55, and 60 ms. The bandwidth was varied for each echo time to the optimal value yielding the best signal-to-noise ratio. Phase encoding was chosen in the anterior–posterior direction because the artifact behavior is often more favorable in this direction. However, phase encoding in the left–right direction would allow for a rectangular FOV and matrix, and hence, a further reduction in measurement times. Fat suppression was achieved using spectral presaturation with inversion recovery (SPIR).

A 3-D high-resolution T1-weighted image was acquired for anatomical reference, using a multi-shot turbo gradient echo with a turbo factor of 32, a 256 × 256 matrix, FOV of 290 mm, slice thickness of 3 mm, TE = 2.30 ms, TR = 20 ms, and a flip angle of 20°.

### Data postprocessing

Native fMRI data was collected in DICOM, and converted to the IMG format for postprocessing.

All data underwent identical analysis with Statistical Parametric Mapping 99 (SPM 99; Wellcome Department of Cognitive Neurology, London, UK)[24] (see also http://www.fil.ion.ucl.ac.uk/spm)

The echo-planar images were realigned using a rigid body transformation to the first volume of the time series for each subject. After this, data were spatially smoothed with a Gaussian filter (FWHM 5 × 5 × 5mm), and spatially normalized. The box-car designed the task was convolved with a hemodynamic response function was used.

T statistics were calculated for each voxel, and *P* < 0.001 was considered to be a statistically significant threshold for significantly activated areas that were correlated for multiple comparisons. The average and maximal BOLD signal changes were calculated for each subject in the motor area M1 for each echo time used.

The average of the maximal BOLD signal changes for all the subjects was also calculated for each echo time. Activation maps were calculated and overlaid on the BOLD single-shot spin-echo EPI with SENSE images. Group analysis of the data was performed, 3D rendering maps were calculated, and the average volume of the activated area M1 was determined for the echo times studied for all subjects.

## Results and Discussion

Brain activations in the motor-sensory cortices were robustly and consistently detected in BOLD images in all subjects. Figure 1a shows a typical example of the BOLD activation maps obtained from three single-shot SE-EPI with SENSE at 3 Tesla with echo times of 35, 45, and 55 ms overlaid on their corresponding BOLD images. Activations in the primary motor-sensory cortex and the supplementary motor area are clearly visible in the maps. More activated voxels were detected in the BOLD maps with higher echo times. Consistent with previous studies, this indicates that BOLD imaging using with appropriate T*_2_ weighting has higher sensitivity in detecting brain activations.[26–28]

**Figure 1a:**
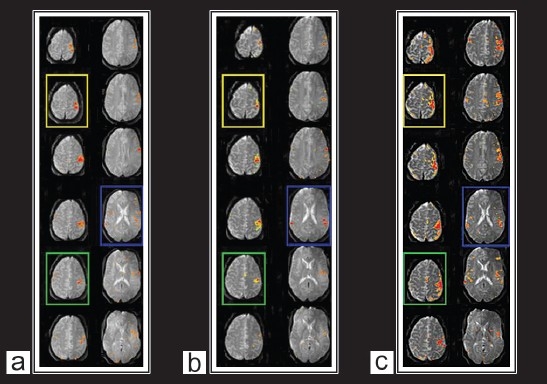
Activation maps of the motor cortex overplayed on example images from single-shot spin-echo EPI series with SENSE (a) TE = 35 ms, (b) TE = 45 ms, (c) TE = 55 ms

Comparing activation maps for the scans corresponding to the three echo times [Figure 1a], larger activation areas were identified in the images obtained at a higher echo time (TE = 55) due to their longer effective TE, whereas more specific activations were detected in the BOLD images obtained at lower echo times.

Time courses of the BOLD signals in the activated areas from the same subject and the same echo times are shown in [Figure 1b]. They show consistent and effective changes in the BOLD signals in the activated area over time, thus fitting the paradigm design of the functional task.

**Figure 1b:**
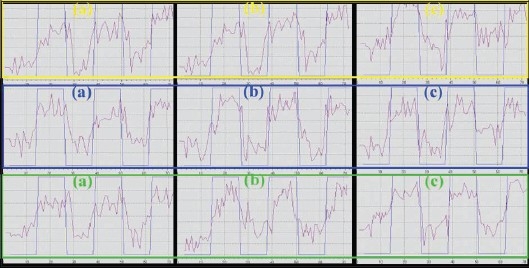
The time course of BOLD signal change in the activated motor cortex corresponding to color boxes in Figure 1a. (a) TE = 35 ms, (b) TE = 45 ms, (c) TE = 55 ms

**Figure 2:**
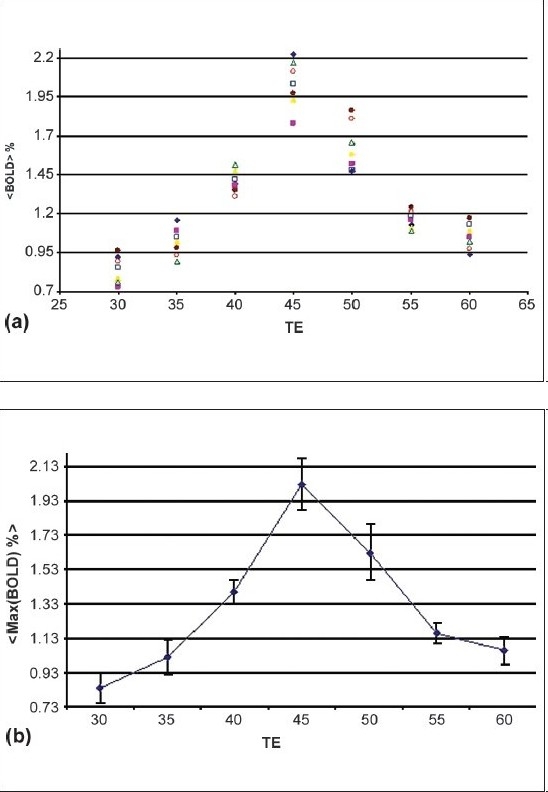
(a) Individual average percentage signal change in M1 for seven subjects as a function of echo time, (b) Maximal percentage signal change attributed to the BOLD effect in M1 averaged over seven subjects as a function of echo time

BOLD signal changes are larger in BOLD images corresponding to echo times 40, 45, and 50 ms with averages of 1.4, 2.1 (the largest change), and 1.66%, respectively; indicating stronger T*_2_-weighting in the former three scans [Figure 2a]. Also shown in the figure is the fluctuation of the BOLD signals, implying a better contrast-to-noise ratio and hence, better detection of brain activation for BOLD.

Figure 2b illustrates the BOLD activation maps and the average BOLD signal change; the maximal BOLD signal changes as a function of the echo times at lower and higher echo times, are low and very similar [Figure 2a and 1b].

**Figure 3:**
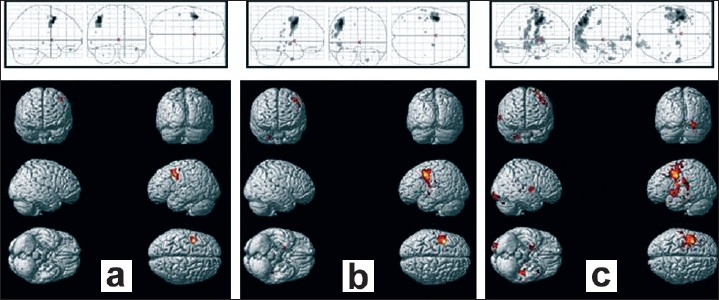
Rendering on a standard brain of activation averaged over seven volunteers, and projections in the stereotactic Tailarach space. The sets correspond respectively to (a) TE = 35 ms, (b) TE = 45 ms, (c) TE = 55 ms

The number of activated voxels due to BOLD signal changes, and activation maps obtained by single-shot SE-SENSE scans for all subjects are detailed in [Table 1 and Figure 3] respectively. An average of 196 ± 29 activated voxels were detected at TE = 55 ms, which is significantly more than the 117 ± 29 voxels detected at TE = 45 ms, and the 81 ± 13 voxels detected at TE = 35 ms.

**Table 1:** Average number of the activated voxels obtained through group analysis of seven healthy subjects

*Echo time in ms*	*Mean size of the activated motor area (M1) expressed in number of voxels*	*Standard error*
35	81	13
45	117	18
55	196	29

This difference in the sizes of the activated areas in the motor area may be explained, at least partially, by the following arguments. First, the echo time is still-defined for single-shot SE-EPI scans with significant durations of the acquisition window but the “effective” TE is actually varied. The TE difference might be less than what it intuitively appears to be (10*vs* 30 ms). Second, although the activation experiments were performed in brain areas with relatively fewer susceptibility gradients, field inhomogeneity always existed due to imperfect shimming and other reasons, particularly at high fields, which could degrade the image quality. As the single-shot EPI sequence is relatively sensitive to field inhomogeneity, more artifacts (*e.g.*, signal attenuation and geometrical distortion) associated with the imperfect field, would present in the BOLD images, leading to reduced SNR.

Finally, both maximal and average signal changes and peak BOLD signals occurred at an echo time of 45 ms [Figure 2a and 2b]. At a very short echo time (TE = 30 ms), the sequence was still sensitive to BOLD effects whereas at higher echo times (TE = 60 ms), a larger volume of activation was seen in spite of a smaller BOLD effect [Figure 2a, 2b and 3]. At all the echo times studied, susceptibility artifacts did not destroy the BOLD signal [Figure 1a and 1b]. Quadrature ghost artifacts that are associated with higher field strengths [Figure 1] and which are typically seen in single-shot EPI, were not visible and, the distribution of activation in the motor, premotor, and sensory regions [Figure 1a and 3] was consistent with that reported in previously reported studies for this motor task.[29,30]

## Conclusion

We have found that single-shot spin-echo echo planar imaging using SENSE at high magnetic fields is a robust algorithm for obtaining functional maps of neuronal activity in the motor system, but it remains sensitive to the echo time used. Susceptibility artifacts associated with higher field fMRI are reduced by the use of SENSE. With the use of appropriate imaging parameters, single-shot SE-EPI with SENSE at higher field systems produces robust maps of functional activity with reduced susceptibility artifacts and better localization of the BOLD signal in the cortical microvascular bed.

We have studied single-shot SE-EPI with SENSE scans in the context of their sensitivity to BOLD signals and their vulnerability to susceptibility-induced artifacts at 3 Tesla. BOLD imaging can be achieved using single-shot SE-EPI with SENSE scanning, with improved BOLD contrast and reduced susceptibility artifacts. Functional experiments with sensorimotor activation in normal subjects demonstrated the advantages of single-shot SE-EPI with SENSE scanning.
